# Site-specific selection reveals selective constraints and functionality of tumor somatic mtDNA mutations

**DOI:** 10.1186/s13046-017-0638-6

**Published:** 2017-11-28

**Authors:** Deyang Li, Xiaohong Du, Xu Guo, Lei Zhan, Xin Li, Chun Yin, Cheng Chen, Mingkun Li, Bingshan Li, Hushan Yang, Jinliang Xing

**Affiliations:** 1 State Key Laboratory of Cancer Biology and Experimental Teaching Center of Basic Medicine, Fourth Military Medical University, 169 Changle West Road, Xi’an, 710032 China; 2 Department of Gastroenterology, Second Affiliated Hospital of Harbin Medical University, Harbin, 150086 China; 3 Fondation Mérieux, 69002 Lyon, France; 4 Center for Human Genetics Research, Department of Molecular Physiology and Biophysics, Vanderbilt University, Nashville, TN 37232 USA; 5 Division of Population Science, Department of Medical Oncology, Sidney Kimmel Cancer Center, Thomas Jefferson University, Philadelphia, PA 19107 USA

## Abstract

**Background:**

Previous studies have indicated that tumor mitochondrial DNA (mtDNA) mutations are primarily shaped by relaxed negative selection, which is contradictory to the critical roles of mtDNA mutations in tumorigenesis. Therefore, we hypothesized that site-specific selection may influence tumor mtDNA mutations.

**Methods:**

To test our hypothesis, we developed the largest collection of tumor mtDNA mutations to date and evaluated how natural selection shaped mtDNA mutation patterns.

**Results:**

Our data demonstrated that both positive and negative selections acted on specific positions or functional units of tumor mtDNAs, although the landscape of these mutations was consistent with the relaxation of negative selection. In particular, mutation rate (mutation number in a region/region bp length) in complex V and tRNA coding regions, especially in ATP8 within complex V and in loop and variable regions within tRNA, were significantly lower than those in other regions. While the mutation rate of most codons and amino acids were consistent with the expectation under neutrality, several codons and amino acids had significantly different rates. Moreover, the mutations under selection were enriched for changes that are predicted to be deleterious, further supporting the evolutionary constraints on these regions.

**Conclusion:**

These results indicate the existence of site-specific selection and imply the important role of the mtDNA mutations at some specific sites in tumor development.

**Electronic supplementary material:**

The online version of this article (10.1186/s13046-017-0638-6) contains supplementary material, which is available to authorized users.

## Background

The human mitochondrial genome is an about 16.5kbp circular mitochondrial DNA (mtDNA) that harbors a control region of 1.1kbp and 37 genes that encode 2 rRNA, 22 tRNA, and 13 proteins of oxidative phosphorylation. Among them, 7 genes encode subunits of complex I (*ND1–6* and *ND4L*); 1 gene encodes a subunit of complex III (*CYTB*); 3 genes encode subunits of complex IV (*COX1–3*); and 2 genes encode subunits of complex V (*ATP6* and *ATP8*) [1]. Due to the lack of protective histones and efficient DNA repair system, the mutation frequency of mtDNA is about 10 times higher than that of nuclear DNA [2]. In the past decade, high-frequency somatic mtDNA mutations have been reported in a variety of diseases, especially in cancers [3, 4].

A series of previous studies established a connection between mitochondrial dysfunctions caused by mtDNA mutations and tumor development and metastasis [5–10]. For example, Cruz-Bermúdez et al. showed that cybrids containing mutations in *ND1* (m.3460G > A), *ND4* (m.11778G > A) and *ND6* (m.14484 T > C) of mtDNA exhibit the enhanced tumorigenicity [5]; Ishikawa et al. revealed that the G13997A and 13885insC mutations in the coding region of *ND6* gene confer an increased metastatic potential in mouse P29 cell line that originated from Lewis lung carcinoma [6].

On the other hand, however, a handful of studies indicated that mtDNA mutations in tumors are primarily shaped by the relaxation of negative selection relative to germline, rather than positive selection [11–13]. For example, Coller et al. reported that random processes are sufficient to explain the incidence of homoplasmic mtDNA mutations in human tumors without the need of positive selection pressure [11]. Stafford et al. concluded that the relaxation of negative selection shapes mtDNA mutation landscapes in cancer cells, as evidenced by the higher ratio of nonsynonymous to synonymous mutations among these mutations [12]. Michele Vidone et al. extracted mtDNA reads from WGS and WES data of glioblastoma multiforme with MToolBox [14] and found that the large majority of mtDNA mutations does not pass the prioritization filters and only a relatively limited burden of pathogenic mutations, which did not appear to determine a general impairment of the respiratory chain, is indeed carried by GBM [15]. Consistently, Liu et al. [13] reported that mtDNA mutations in esophageal cancer and other tumors are most likely shaped by the relaxation of negative selection, and pointed out that the observation of some level of positive selections [16] is likely due to sample mix-up or contamination of exogenous DNA. Recently, two groups [17, 18] reported that most of missense mutations of mtDNA do not have distinct physiological advantages, indicating that these mutations are not under positive selection. In addition, several studies also concluded that mtDNA mutations are not related to the developments of most cases of lung cancer [19] and head and neck squamous cell carcinoma [20]. All of these lines of evidence seem to be contradictory to the critical roles of mtDNA mutations in tumor development, leaving unclear whether somatic mtDNA mutations are drivers, or just bystanders, of tumor development.

The discrepancies among the findings of these various studies may partially be attributed to their limitations, such as small patient populations, incapacity of sequencing methods to detect low level of heteroplasmy, and neglect of replication-mediated mutation frequency bias. Another possibility is that these evolution studies have mainly focused on the overall level of mtDNA mutations, but not on their level in specific regions or individual positions. In contrast, functional studies have mostly focused on individual mutations.

To shed more light on the subject, we aimed to explore whether site-specific natural selection might affect tumor somatic mtDNA mutations and, more importantly, their functions. We performed a systematic and comprehensive exploration of mtDNA mutations in various cancer types, and we developed the largest collection of mtDNA mutations in cancer to date using next generation sequencing (NGS) data generated in various sources, including The Cancer Genome Atlas (TCGA), International Cancer Genome Consortium (ICGC), other publications, as well as our own lab. We analyzed the mutations across the mitochondrion genome as well as in different genomic regions and functional units (e.g., gene domains, codons, amino acids) to evaluate how natural selection shapes mtDNA mutation patterns both globally and locally. These comprehensive analyses of mtDNA mutations, conducted at an unprecedented scale, shed important novel insights on the roles of mtDNA mutations in tumorigenesis.

## Methods

### Data of somatic tumor mtDNA mutations from public resources and our lab

We identified 5423 somatic mtDNA mutations from whole genome sequencing (WGS) and whole exome sequencing (WES) data reported in literature [17, 18, 21], as well as in TCGA (https://portal.gdc.cancer.gov/exploration?searchTableTab=mutations&ssmsTable_size=100) and ICGC (https://dcc.icgc.org/repositories) datasets. For patients who were present in two or more datasets and sequenced by the same method (WES or WGS), the mutations were intersected; for patients who were present in two or more datasets and sequenced by different methods, the data from WGS were retained. Seven patients with an extremely large number of somatic mutations (≥13) were excluded from further analyses.

In addition, we used mtDNA capture sequencing approach to identify 104 somatic mutations from 54 colon cancer patients, 357 somatic mutations from 143 hepatocellular carcinoma (HCC) patients, and 36 somatic mutations from 15 kidney cancer patients. All these mutations derived from tumor tissue in our lab were verified by parallel sequencing of the non-tumor tissue of the same patient. Considering the error rate of the Illumina sequencing platform, only the somatic mutations with ≥2% of variant allele fractions were counted.

Finally, we generated a combined dataset containing 26 tumor types, 3277 patients, and 5920 mutations, the most comprehensive tumor mtDNA mutation dataset to date (Additional file 1: Table S1 and Additional file 2: Table S2). Among them, there were 2465 mutation records with heteroplasmic level data from three publications. The heteroplasmic levels of 497 mutations from our lab data were calculated as the ratio of the mutant reads in all reads that cover the mutation positions. All mutations were annotated using Annovar software [22].

### Germline variation data in natural human population

Germline variation data containing 4322 mtDNA single base substitutions in the natural human population was extracted from phylotree (Build 17, 18 Feb 2016) [23]. The htm file of mtDNA tree Build 17 containing all mtDNA variations among populations to date was downloaded (http://www.phylotree.org/builds/mtDNA_tree_Build_17.zip) and the base substitution information was extracted using a homemade python script. (Additional file 3: Table S3).

### Recurrent rate analysis

Recurrent rate of mutation in a specific region was determined by formula:

$F\kern0.5em =\kern0.5em \frac{\varSigma R}{L}$, in which F stands for mutation rate, R for recurrent times of mutations in a position, and L for the length (bp) of regions. If the R value of a mutation was greater than 1 in patient group, this mutation was considered recurrent mutation.

### Prediction of functional impacts of mutations in mRNA and tRNA coding regions

The functional impacts of mutations in mRNA coding regions were predicted according to Mitimpact 2.5 [24], and were classified as high, medium, low, or neutral impact. The functional impacts of mutations in tRNA coding regions were predicted, as described by Kondrashov et al. [18], and were classified as either benign or deleterious.

### Simulation of mtDNA mutations

**Fig. 1:**
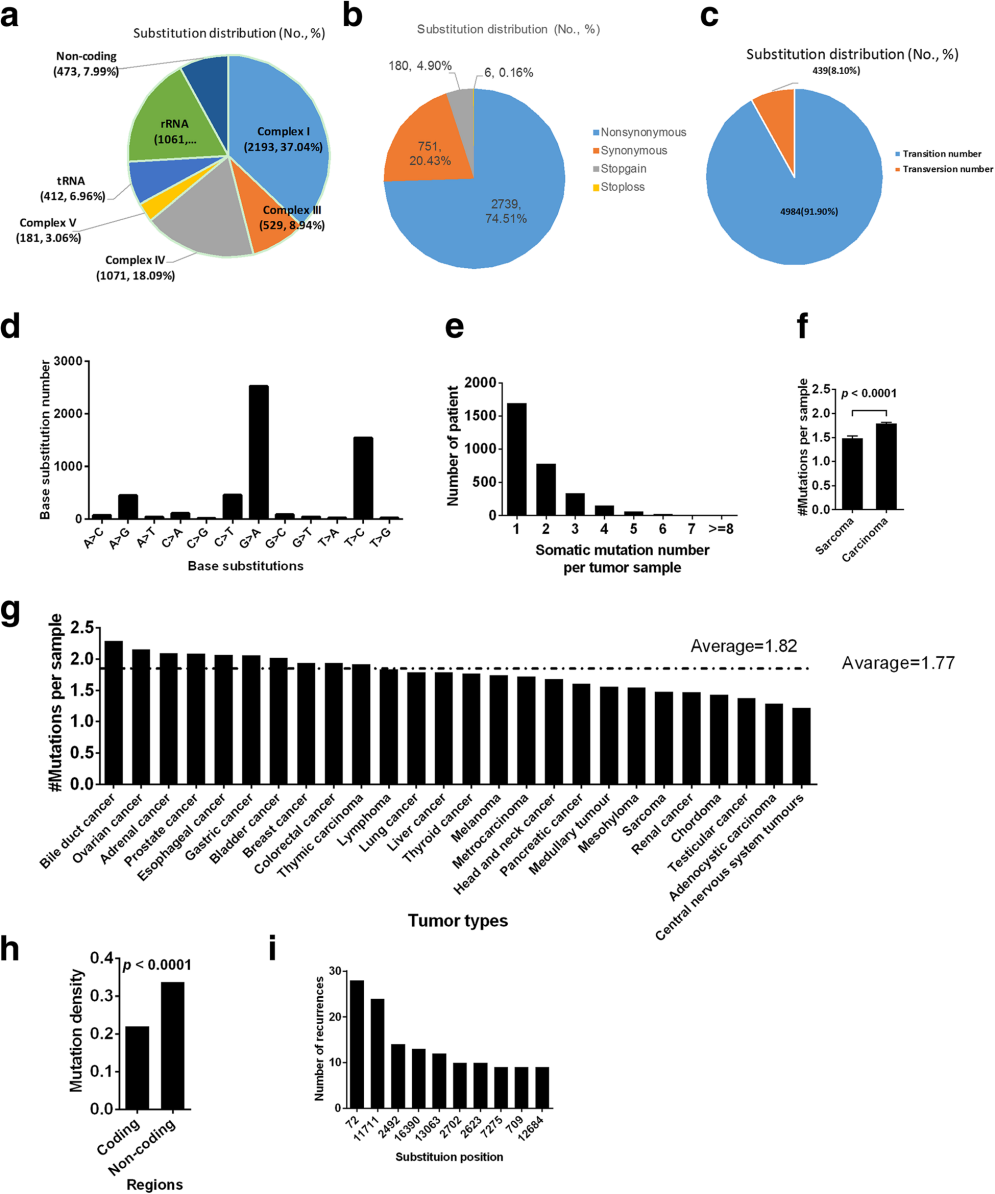
Characteristics of 5920 tumor somatic mtDNA single base mutations. **a** Number and proportion of mutations in different regions. **b** Number and proportion of nonsynonymous mutations, synonymous mutations, stoploss mutations and stopgain mutations. **c** Number and proportion of transition and transversion. **d** Number of base substitutions. **e** Number of somatic mutations in a tumor patient. **f** Average number of somatic mutations in carcinoma and sarcoma. **g** Average number of somatic mutations per patient across 26 tumor types. **h** Mutation densities (mutation number in a region/region bp length) in coding regions and non-coding regions. **i** Number of the top 10 recurrent mutations. Mean ± SEM, t test was used

We now describe a process to simulate mtDNA mutations under neutrality as a reference. Since it is impossible to know the mutation rate of each base under neutrality, we assumed that the vast majority of the mtDNA mutations in tumors were derived under neutrality, and selection plays a negligible role in shaping the overall mutation pattern. Under this assumption, we utilized the mutations in the large-scale dataset we collected to calibrate neutral mutation rates. Specially, we calculated mutation frequency of each of the possible nucleotide base substitutions (12 types in total) based on the observed base substitutions in this dataset. That is, for each of the 12 base substitutions, we counted the number of occurrences observed in this dataset, to represent the mutant frequency under neutrality; such estimates are expected to be robust given the large size of the collected mutation dataset. In simulating a mutation dataset under neutrality, we first stored all the calibrated base substitution frequencies in a file (Additional file 4: Table S4), and then used a homemade python script to randomly simulate 12 types of base substitutions, according to the mutation frequencies stored in this file (Fig. 1b). The data thus simulated have the same distribution of mutations observed in the large-scale mutation dataset we collected, representing mutations derived under neutrality. We repeated this process N times to generate random replicates of mutations, and used the simulated datasets as a reference of neutrality to compare against the observed mutations in a dataset. Simulating scripts are available from https://github.com/gadbee/Bioinformatics.

### Statistical analysis

All statistical tests were performed using Graphpad Prism software (GraphPad Software, Inc.). Comparisons between groups with continuous variables and categorical variables were performed using Student’s t test, and then ANOVA and Chi Square test. Pearson’s correlation coefficient was used to evaluate the correlations among related variables. All *p*-values were two-tailed. Residual was calculated by the difference between simulated data and observed data of a mutant codon or an amino acid. Standardized residual was calculated by formula: ${\updelta}^{\ast }=\left(\updelta -\overline{\updelta}\right)/\upsigma$, in which δ* stands for standardized residual, δ for residual of a certain data point, $\overline{\updelta}$ for the average of all residuals, and σ for the standard deviation of all residuals. Standardized residuals greater than 2 were considered significant deviation of observed values from simulated values.

### Ethical statement

The study was approved by the Institutional Review Boards of FMMU and informed written consent was obtained from each patient. The experiment was conducted according to the Declaration of Helsinki.

## Results

### Characteristics of 5920 Single Base mtDNA mutations

A total of 5920 single base mutations from 3277 patients of 26 tumor types (Additional file 1: Figure S1) were collected from two databases (TCGA and ICGC), three previous publications, and our mtDNA sequencing data (see Materials and Methods and Additional file 2: Table S2). Similar to previous report [17], a high consistency among WGS, WES, and mtDNA capture data was observed by comparing the proportion of synonymous and nonsynonymous mutations (Additional file 1: Figure S1B). In addition, we also analyzed the correlation of base substitution numbers or the amino acid substitution number between WGS and WES data (Additional file 1: Figure S1C, D) or between WGS + WES and mtDNA capture data (Additional file 1: Figure S1E, F) and found a high consistency of substitution pattern among the data derived from different sequencing methods. These findings indicated that all data were correctly combined for further analyses. A total of 5920 mutations included 816 synonymous, 2946 nonsynonymous, and 209 stop-gain/stop-loss mutations at mRNA coding regions and 1949 mutations at tRNA, rRNA coding regions and non-coding region. The vast majority (5449, 92.04%) were transitions (Fig. 1a), and most (5447, 92.01%) mutations were located in coding regions (Fig. 1a). The mutations had an obvious bias towards specific base substitutions, with C_H_ > T_H_ followed by T_L_ > C_L_ having a much higher rate than others (Fig. 1b).

Among the 3277 tumors that harbored at least one somatic mutation, 1500 (45.77%) had multiple mutations, ranging from 2 to 8 (Fig. 1c). The mutation rate (mutation number in a region/region bp length) in non-coding regions were significantly higher than those in coding regions (Fig. 1d). The average mutation number per patient in different tumor types was 1.82, ranging from 2.29 (bile duct cancer) to 1.22 (central nervous system tumors) (Fig. 1e, left). Interestingly, the average mutation number in sarcomas was significantly lower than those in carcinomas (Fig. 1e, right). The mutation levels, available for 2962 mutations, ranged from 2% heteroplasmy to 100% homoplasmy. Of the 5920 somatic mutations, more than half (2951, 51.10%) were not registered in the mitomap database updated on 29 Dec 2016 [25].

### The relaxation of negative selection on tumor somatic mtDNA Single Base mutations

**Fig. 2:**
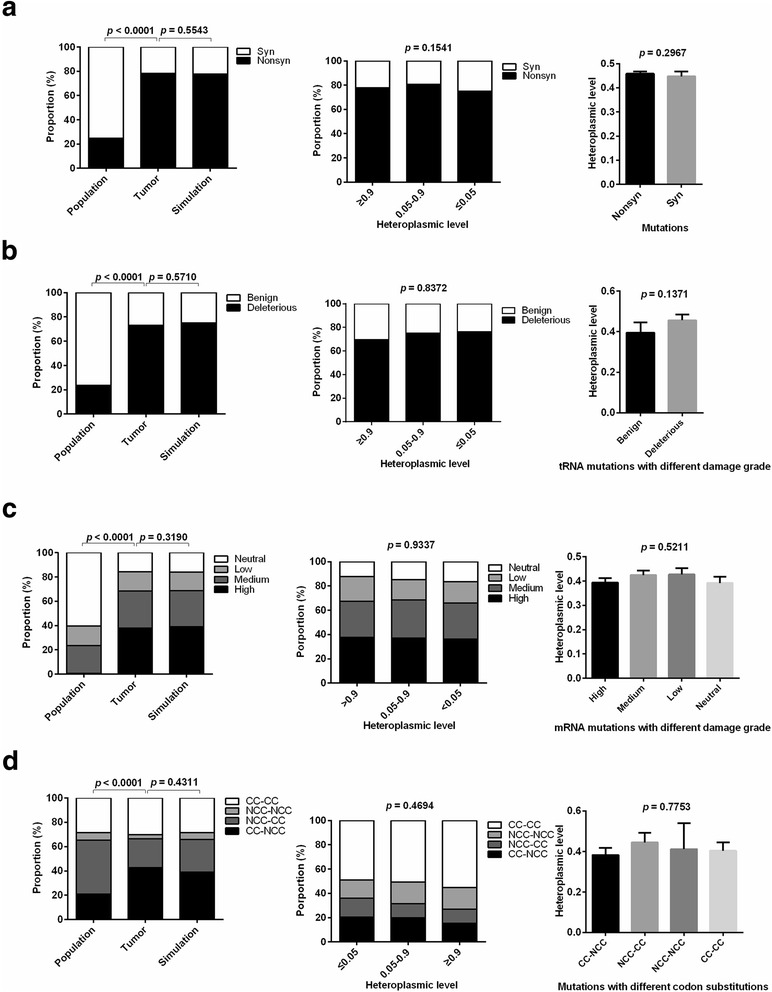
The relaxation of negative selection on majority of tumor somatic mtDNA single base mutations. **a**, **b**, **c**, **d** Proportion of synonymous and nonsynonymous single base mutations in mRNA-coding regions, benign and deleterious in tRNA coding regions, mutations with different damage grades in mRNA coding regions, those resulting in different codon substitutions in population, tumor and simulation data (Left); Proportion only in tumor data stratified by heteroplasmic levels (Middle); Average heteroplasmic level in tumor data (Right). CC: codons completely complementary to anti-codons. NCC: codons not completely complementary to anti-codons. Left and middle: chi-square test was used. Right (**a**, **b**): t test was used; Right (**c**, **d**): ANOVA was used

To evaluate the effects of natural selection on single base mutations, mutation patterns were analyzed in general population, tumor, and simulation data. Compared with general population data, tumor data showed a higher proportion of nonsynonymous mutations in mRNA-coding regions, more deleterious mutations in tRNA coding regions, higher impact on protein function, and more codons that cannot be completely complementary with anticodon (Fig. 2a-d, left panel). These phenomena were considered as a result of positive selection in previous studies [17]. Alternatively, another plausible model has been suggested that these tumor mutation patterns were shaped by mutation rate bias and relaxation of negative selection [13, 17]. To further investigate whether the observed mutation patterns in the datasets that we developed are consistent with the relaxation of selection, we generated simulation data of mtDNA mutations under neutrality based on the mutation rate bias shown in Fig. 1b (see Methods). No significant differences existed between the observed mutations and the simulated data, supporting the effects of the relaxation of selection on mtDNA mutations at the genome scale (left panel of Fig. 2 a-d).

To rule out the possibility that mutations with different heteroplasmic levels are under different selective constraints, we stratified the analyses by heteroplasmic levels (≤0.05, 0.05–0.9, ≥0.9) and found that the same conclusion holds regardless of the heteroplasmy levels (Fig. 2 a-d, middle panel). Furthermore, we found that heteroplasmic levels were not significantly different between synonymous and nonsynonymous mutations, between deleterious and benign mutations in tRNA coding regions, among mutations with different damage grades in mRNA coding regions, and among mutations resulting in different codon substitutions (Fig. 2a-d, right panel). Taken together, our results indicate that single base mutations at the genome scale are not restricted by natural selection, which is consistent with previous reports [17, 18].

### Functional unit-specific selection on tumor somatic mtDNA mutation

**Fig. 3:**
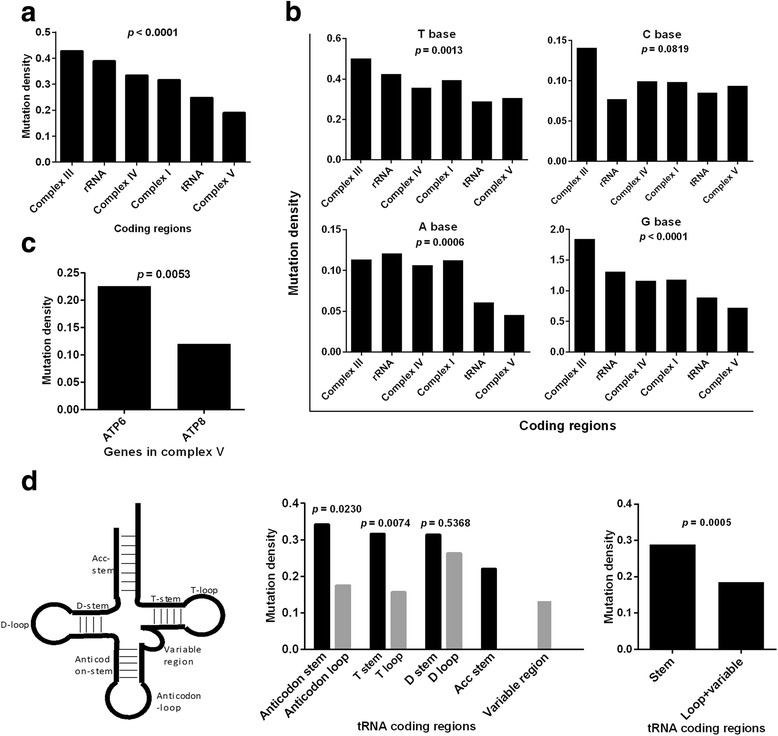
Functional unit-specific selection on tumor somatic mtDNA mutations in coding regions. **a** Mutation densities (mutation number in a region/region bp length) in different coding regions. **b** Mutation densities of T, C, A, G bases in different coding regions. **c** Mutation densities in *ATP6* and *ATP8* genes. **d** Mutation densities in functional units of tRNA coding regions. tRNA functional units were shown (Left). Chi-square test was used

Previous studies demonstrated strong negative selection on truncating mtDNA mutations and tRNA anticodons [17, 18]. To further explore the effects of functional unit-specific natural selection on tumor mtDNA mutations, we compared the mutation rate (mutation number in a region/region bp length) in different mtDNA coding regions. Interestingly, we found that the mutation densities in complex V and tRNA coding regions were significantly lower than those in other regions (*p* < 0.0001, Fig. 3a). Considering the different mutation rates of 4 bases (Fig. 1b), the stratified analyses were performed. Our results indicated that the mutation rates of complex V and tRNA coding regions were the lowest among 6 subgroups when stratified by A, G and T bases (Fig. 3b). We further explored the mutation densities within the complex V and tRNA regions and found that *ATP8* has a lower mutation density than *ATP6* in complex V region (*p* = 0.0053, Fig. 3c). The mutation densities in the loop/variable regions in tRNA were significantly lower than those in the stem regions (*p* = 0.0005, Fig. 3d, middle and right panels).

### Site-preferred recurrence of tumor somatic mtDNA mutations

**Fig. 4:**
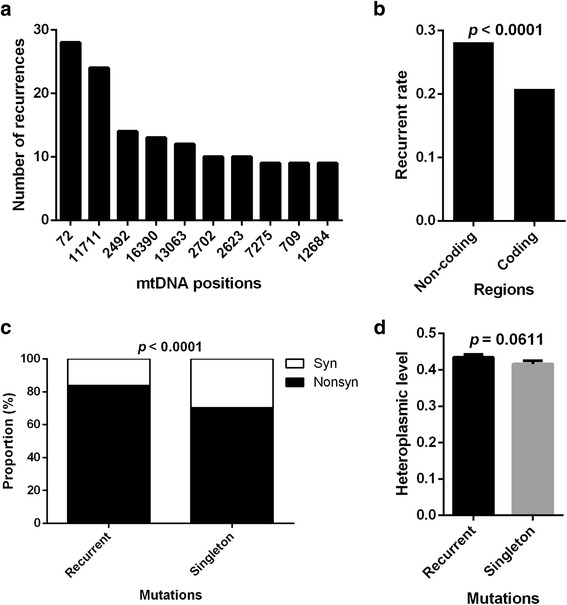
Site-preferred recurrence of tumor somatic mtDNA mutations. **a** Recurrent times of top 10 mtDNA Positions. **b** Recurrent mutation rate, which was defined as the total recurrent times of mutations divided by bp length of region, in non-coding and coding regions. Chi-square test was used. **c** The proportion of nonsynonymous and synonymous mutations in recurrent mutations and singletons. **d** The heteroplasmic level of recurrent mutations and singletons

A total of 5920 mutations occurred at 3590 distinct positions, among which 2384 were singletons and 3536 (59.73%) were recurrent. The recurrent mutations can be collapsed onto 1206 mtDNA positions with recurrence rate (i.e., number of patients carrying the same mutation) ranging from 2 to 28. The 10 most recurrent mutation positions were listed in Fig. 4a. Furthermore, we found that non-coding regions had a higher probability of recurrent mutations than coding regions (*p* < 0.0001, Fig. 4b). The proportion of nonsynonymous mutations was higher in recurrent mutations than in singletons (p < 0.0001, Fig. 4c). The heteroplasmic levels of recurrent mutations tend to be higher than those of singletons, although the differences were not significant (*p* = 0.0611, Fig. 4d). These findings indicate that the recurrence of tumor somatic mtDNA mutations is site-preferred, especially in non-coding region, and suggested the presence of positive selection of mtDNA mutations in tumors.

### Codon-specific selection on tumor somatic mtDNA mutations

**Fig. 5:**
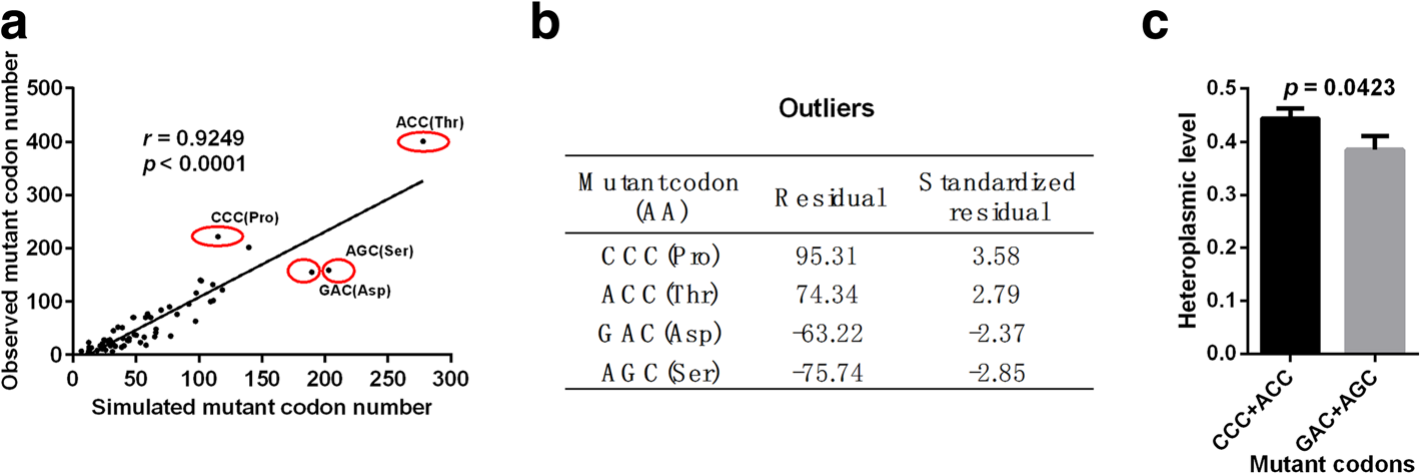
Codon-specific selection on tumor somatic mtDNA mutations. **a** The correlation between numbers of mutation-caused codon from observed and simulated data. Red circles showed 4 codons which were significantly deviated from trend line. **b** Residuals and standardized residuals of four outliers in (**a**) were shown. **c** Heteroplasmic level of mutation-caused codons. Mean ± SEM, t test was used

Given that codons are the most fundamental units for protein coding, we are interested in learning whether specific mutation-caused codons are favorably or unfavorably selected. To this end, the number of each codon generated by base mutation from simulated data was analyzed and compared with that from the observed data. As expected, the results from simulated and observed data were highly correlated (*r* = 0.9249, p < 0.0001, Fig. 5a), also indicating that the overall codon mutations are shaped by the relaxation of negative selection. However, several specific codon mutations significantly deviated from the trend line (i.e., above or below) by more than 2 standardized residuals. Among them, the observed numbers of mutant codon CCC (Pro) and ACC (Thr) were significantly higher than the simulated values (standardized residuals of 3.58 and 2.79, respectively), suggesting that positive selection might act on these specific mutant codons. In contrast, the observed numbers of mutant codon GAC (Asp) and AGC (Ser) were significantly lower than the simulated values (standardized residuals of −2.37 and −2.85, respectively), implying the possibility of negative selection of these mutant codons (Fig. 5a, b). To investigate the robustness of the analysis, we carried out the same simulation 10 times and observed consistent patterns in all simulations (data not shown). To exclude the possibility that the outliers were influenced by the local sequence context of mutations, we re-analyzed the site-specific selection by examining the bases immediately 5′ and 3′ to the mutated bases. As shown in Additional file 1: Figure S3, significant correlation between observed and simulated mutant triplet-bases was observed (*r* = 0.9836, *P* < 0.0001). Moreover, three triplet-bases CAC, CAA and GAC with above −2 or 2 of standardized residuals were identified, suggesting that local sequence context may contribute to evolutional selection of specific mutations, although the underlying mechanism is largely unknown. Further analysis indicated that these three triplet-bases were not related with the selected codons shown in Fig. 5a. Specially, triplet-bases GAC in Additional file 1: Figure S3 and codon GAC in Fig. 5a were located on the different sides of trendline. Our results suggest that the outliers in Fig. 5a may not be determined by the context sequence. In addition, we also found that the average heteroplasmic levels of the base mutations in codon CCC and ACC were significantly higher than those in codon GAC and AGC (*p* = 0.0423, Fig. 5c), further supporting the existence of codon-specific selection on tumor mtDNA mutations.

### Amino acid-specific selection on tumor somatic mtDNA mutations

**Fig. 6:**
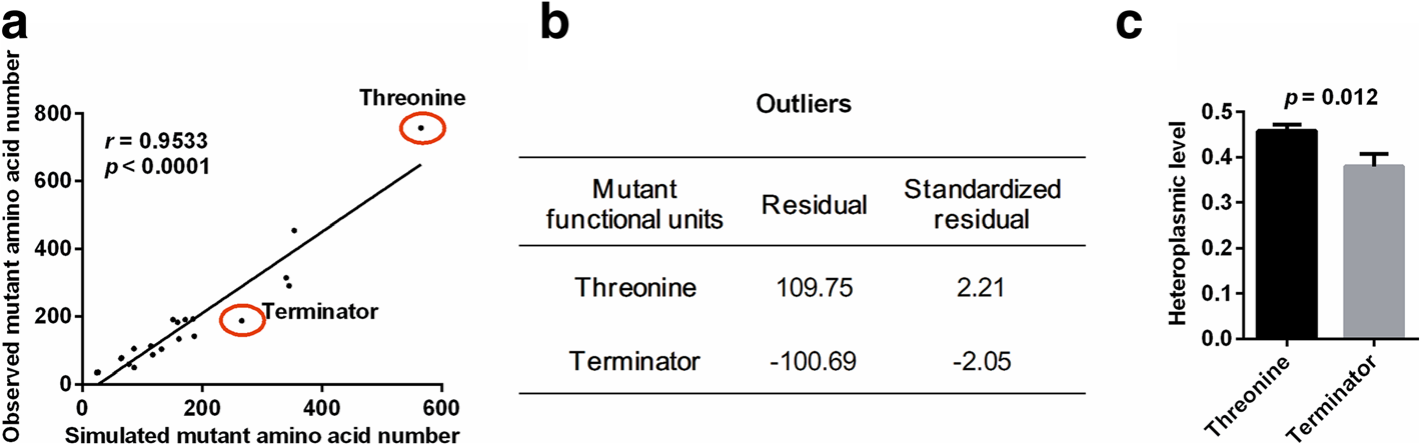
Amino acid-specific selection on tumor somatic mtDNA mutations. **a** The correlation between the number of mutation-caused amino acid from observed and simulated data. Red circles showed the mutation-caused threonine and terminator deviated from trend line. **b** Two outliers in (**a**). Standardized residuals indicate threonine and terminator are selected. **c** Heteroplasmic level of mutation-caused threonine and Terminator. Mean ± SEM, t test was used

We further investigated the site-specific selection of mtDNA mutations at the amino acid level. Similarly, the number of each amino acid generated by mutant codons from observed or simulated data was determined. Once again, we found that the value from the simulated data was highly correlated with that from the observed data (*r* = 0.9533, *p* < 0.0001, Fig. 6a), further confirming the relaxation of negative selection for overall mutations. Nevertheless, the mutant amino acid Thr was significantly above the trend line (standardized residual of 2.21) and Ter (terminator) was significantly below the trend line (standardized residual of −2.05) (Fig. 6b). In addition, both amino acids Pro and Ser were also above and below trend line, respectively, although the deviations were not significant (standardized residuals of 1.23 and −0.89, respectively). We also observed that the average heteroplasmic level of the base mutations in amino acids Thr was significantly higher than that in Ter (*p* = 0.012, Fig. 6c), which supports the notion that mutant amino acids are under specific selection. Notably, these findings align well with the selection pattern of codon mutations shown in Fig. 5.

### Functionality of tumor somatic mtDNA mutations under site-specific selection

**Fig. 7:**
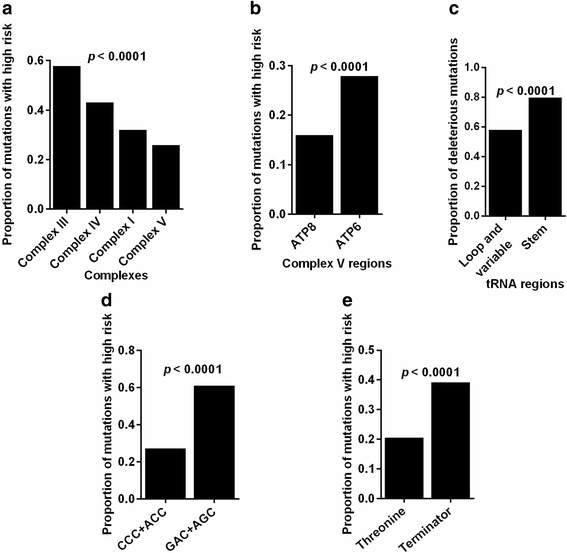
Functionality of tumor somatic mtDNA mutations under site-specific selection. **a** Proportion of mutations with high and medium impact in different coding regions. **b** Proportion of mutations with high impact in *ATP6* and *ATP8* coding regions. **c** Proportion of deleterious mutations in stem or loop and variable regions of tRNA. **d** Proportion of mutations with high impact in mutation-caused codons with significant positive and negative selection. **e** Proportion of mutations with high impact in mutation-caused threonine and terminator. Chi-square test was used

We investigated the predicted functionality of mutations in different genome regions and found that the proportion of mutations predicted to be highly deleterious was significantly different among complex III, IV, I, and V coding regions with the lowest value in complex V (p < 0.0001, Fig. 7a), suggesting the stricter negative selection on complex V, especially in *ATP8* (p < 0.0001, Fig. 7b). We also observed that the proportions of deleterious mutations in loop and variable regions were significantly lower than those in stem regions (p < 0.0001, Fig. 7c), indicating the possibility of stricter negative selection on loop and variable regions of tRNA. Moreover, we observed that the proportions of deleterious mutations in CCC and ACC codons and threonine were significantly greater than those in GAC and AGC codons and terminator, respectively (both p < 0.0001, Fig. 7d and e).

## Discussion

Somatic mtDNA mutations have attracted significant attention because of their high frequencies in cancers [3, 4]. Specific mtDNA mutations have been implicated in tumor development and metastasis [5, 6]. However, an increasing number of recent studies have shown that tumor mtDNA mutations are under relaxed negative selection [13, 17, 18]. While the differing findings of these studies has raised controversy over the existence of specific functional mtDNA mutations, the indications of the relaxation of negative selection on the majority of tumor mtDNA mutations does not completely exclude the possibility of site-specific selection on individual mutations. To resolve these controversies, we developed a comprehensive dataset of tumor mtDNA mutations and systemically analyzed the effects of site-specific selection on these mutations. We found most tumor mtDNA mutations are under the relaxed negative selection, which is highly consistent with previous studies [13, 17, 18]. We also noted that some specific regions are under stricter negative selection than other regions.

We further found that mutations in tRNA and complex V coding regions showed evidence of negative selection, especially in *ATP8* gene coding regions of complex V and loop and variable regions of tRNA. In line with our findings, a previous study reported that *ATP8* gene seems to be protected from the relaxation of negative selection [26, 27]. Moreover, we found that the selective pattern on a region is related with the deleterious risk of mutations. This finding further confirms the fact that mutations in complex V and tRNA are under stricter negative selection, especially in *ATP8* of complex V and variable/loop regions of tRNA, and partially explains the reason of evolutional constrains on these regions. This evolutional constraint implies that the mutations disrupting the normal function of complex V and tRNA may confer an evolutionary disadvantage for tumors that predispose tumor to being negatively selected. Consistently, the important role of complex V in the etiology of tumor is also supported by experimental mouse data. For example, Petros et al. revealed that the prostate cancer cells with mutation T8993G in *ATP6* gene generated larger tumor than wild-type cancer cells [28]. The essential role of the electron transport chain in aspartate synthesis [29] may be the reason that mtDNA mutations, especially those deleterious mutations, are negatively selected. The functional roles of mutations in complex V and tRNA in tumor need in-depth future investigations.

Recurrent mutations were analysed in this study. Generally, the recurrence of mutation in population tends to be the evidence of positive selection [30], which partially support our ratiocination. However, we cannot rule out such a possibility of negative selection on coding regions of mtDNA in tumors, which needs to be further explored in future studies.

Our study also identified the specific codons and amino acids which tend to be under positive or negative selection. Our findings from selected codons and amino acids further supported the existence of site-specific selection of tumor mtDNA mutations and demonstrated that some selected mutations have potentially important functions in tumors. Moreover, our finding that the mutation-caused terminator was negatively selected is consistent with previous reports [17, 18], further supporting the reliability of our findings. Our analyses also showed that those codons that tend to be positively selected have a significantly higher pathogenic risk than those that tend to be negatively selected, and this may be the reason why these mutations are differentially selected. In consideration of the deleterious impact of stop-gain mutations, the negative selection on stop-gain mutations enhanced the important role of mtDNA in tumors. The positive selections of codon CCC and ACC and amino acid Thr may imply the growth advantage they confer to tumor cells and needs to be further explored in the future.

In addition to the functional impact of the mutations in mRNA coding regions, the impacts on the mitochondrial pool of free amino acids may be one of the reasons that some amino acids are differentially selected. Previous study suggests that the concentration of free amino acids in mitochondria is positively correlated with the composition of mtDNA-encoded proteins [31]. Therefore, the mutation-caused composition changes of amino acids in mtDNA-encoded proteins may consume some free amino acids and increase the concentration of other free amino acids, which may influence the concentration of free amino acids and cause the unbalance of free amino acids pool in mitochondria. Considering the important roles of the concentration of free amino acids in tumor cells [29, 32–36], the changes in free amino acid concentration in mitochondria may influence the phenotype of tumor cells. This conjecture is supported by the observation that the codons/amino acids identified to be selected in our study (Asp, Ser, Pro) overlap with the amino acids that are reported to influence the phenotype of tumor cells [34–36]. However, it is largely unknown about the cause-effect relationship between mtDNA mutation and the change of free amino acid concentration. On one hand, it is possible that the state of the amino acid pool may result in some codons/amino acids to be selected for. On the other hand, the impacts of mutation on the mitochondrial pool of free amino acids may be one of the reasons that some amino acids are differentially selected. Therefore, the relationship between the amino acid pool and mtDNA mutation need to be further explored in future study.

## Conclusions

Our results showed that the global landscape of tumor mtDNA mutations is shaped by the relaxation of negative selection, and our detailed analysis also identified regions and functional units that are under site-specific positive or negative selection, suggesting the extensive involvement of natural selection on mtDNA mutations during tumor development. The site-specific selection of tumor mtDNA mutations provides clues to identify driver mtDNA mutations of tumorigenesis and progression. More tumor mtDNA sequencing data and mechanistic experiments are needed to gain further insights into the roles of mtDNA mutations in tumors.

## Additional files

Additional file 1: Figure S1. **Figure S2.** and **Table S1.** (DOCX 833 kb)

Additional file 2: Table S2. Information of 5920 tumor mtDNA base substitutions. (XLSX 488 kb)

Additional file 3: Table S3. Phylotree data annotation. (XLSX 224 kb)

Additional file 4: Table S4. All possible base substitution in mRNA coding regions of mtDNA. (XLSX 1519 kb)
