# Supplementary material for: Site-specific selection reveals selective constraints and functionality of tumor somatic mtDNA mutations
Source: J Exp Clin Cancer Res. 2017 Nov 28;36:168. doi: 10.1186/s13046-017-0638-6 (PMC5704541; doi:10.1186/s13046-017-0638-6)
Supplement: Supplementary file 1 — Figure S2. and Table S1. (DOCX 833 kb) [file 13046_2017_638_MOESM1_ESM.docx]

、

FIG. S1. The consistency among WGS, WES and mtDNA capture data. (A) The number of patient sequenced by different sequencing methods. (B) Proportion of nonsynonymous and synonymous mutations come from different sequencing methods (χ square test). (C) The correlation of base substitution number come from WGS and WES. (D) The correlation of amino acid substitution number come from WGS and WES. (E) The correlation of base substitution number come from WGS+WES and mtDNA capture. (F) The correlation of amino acid substitution number come from WGS+WES and mtDNA capture.

FIG. S2. Tumor types of 3277 patients. Samples sequenced in our lab are mixed with public data.

FIG. S3. The number of triplet-base containing the sequence context of mutation from simulated data and observed data.

*Single-base but not indel mutations were extracted from all datasets.

& WGS: whole genome sequencing; WES: whole exome sequencing.

@Actual number of tumor types were less than the total number of tumor type from all datasets because some tumor types were included in two or more datasets and only one was kept, as well as some tumor types were combined.

# Only patients carrying mtDNA mutation(s) were counted.

% Mutations from the patients which were sequenced two or more times by the same method were intersected; while if the sequencing method is different(WES and WGS), the mutation records from WGS were kept.

$Actual number of patients was less than the total number of patients from all datasets because some patients were included in two or more datasets and only one was kept. 7 patients with excessive number (≥13) of somatic mutation were excluded.

^Actual number of mutations was less than the total number of mutations from all datasets because some mutations were included in two or more datasets and only one was kept.
